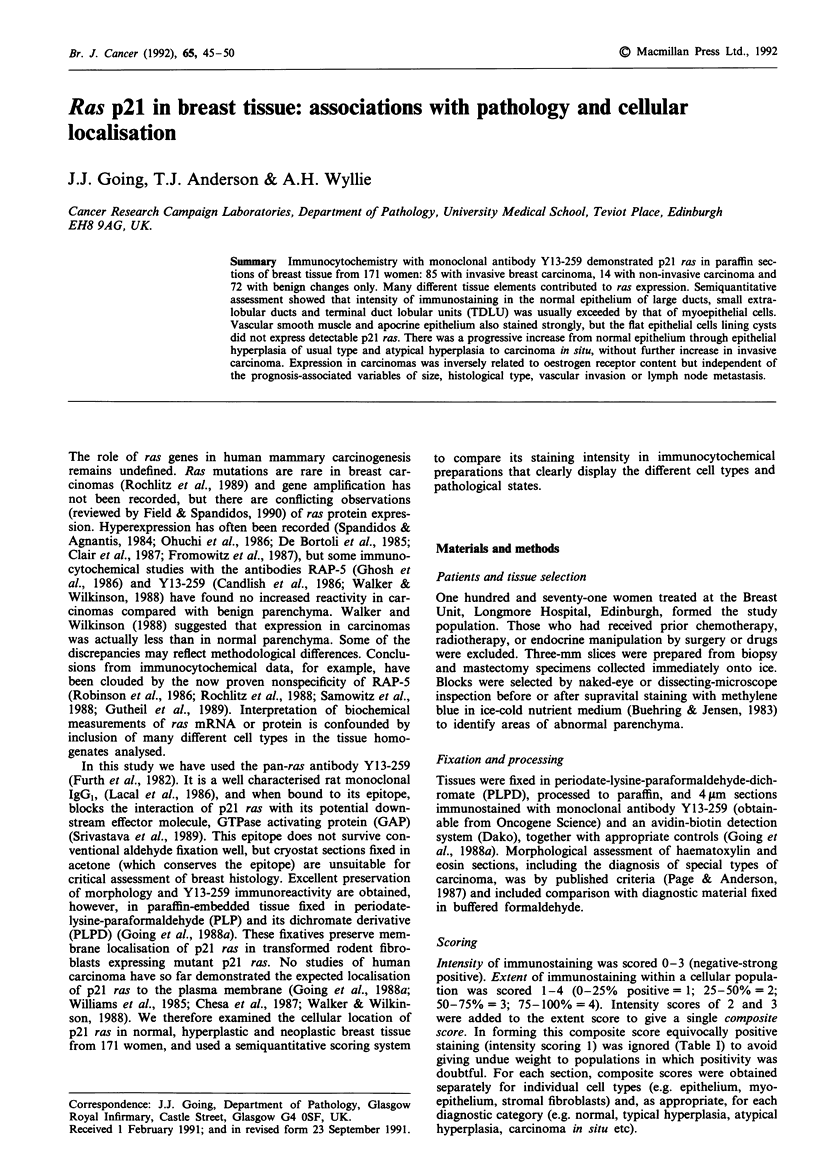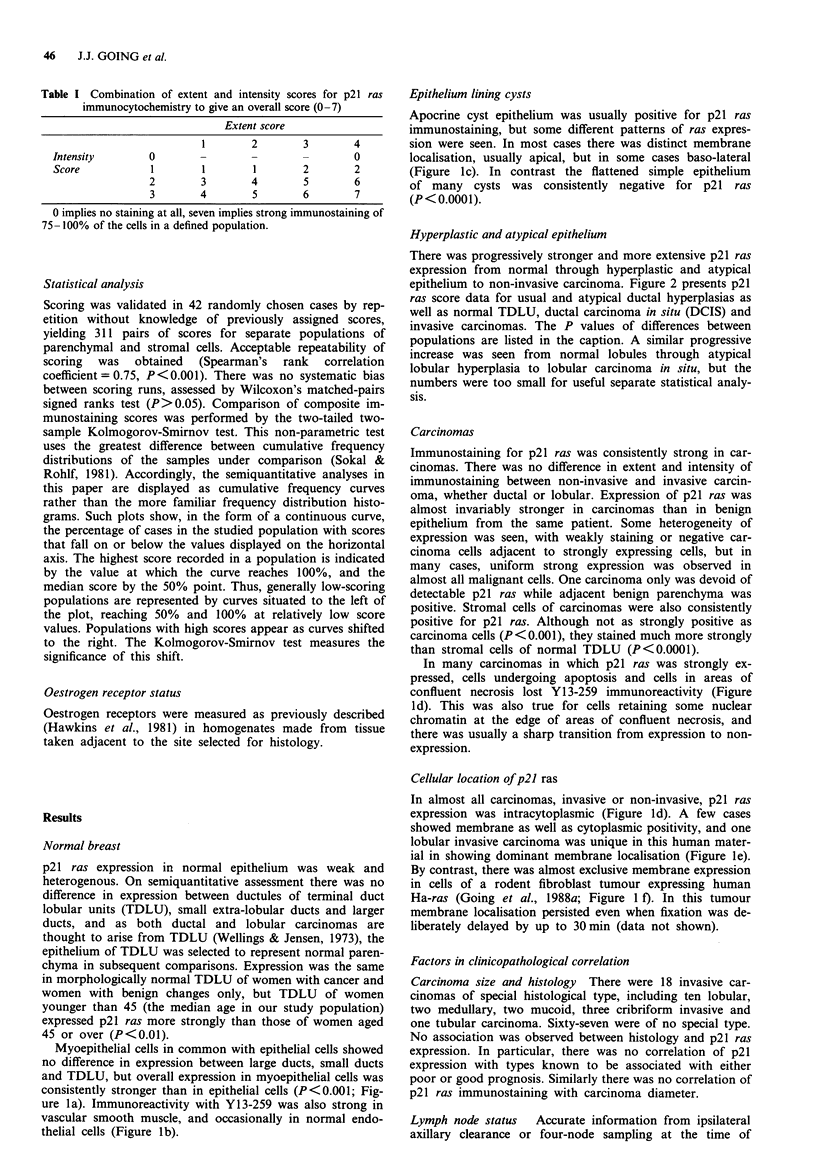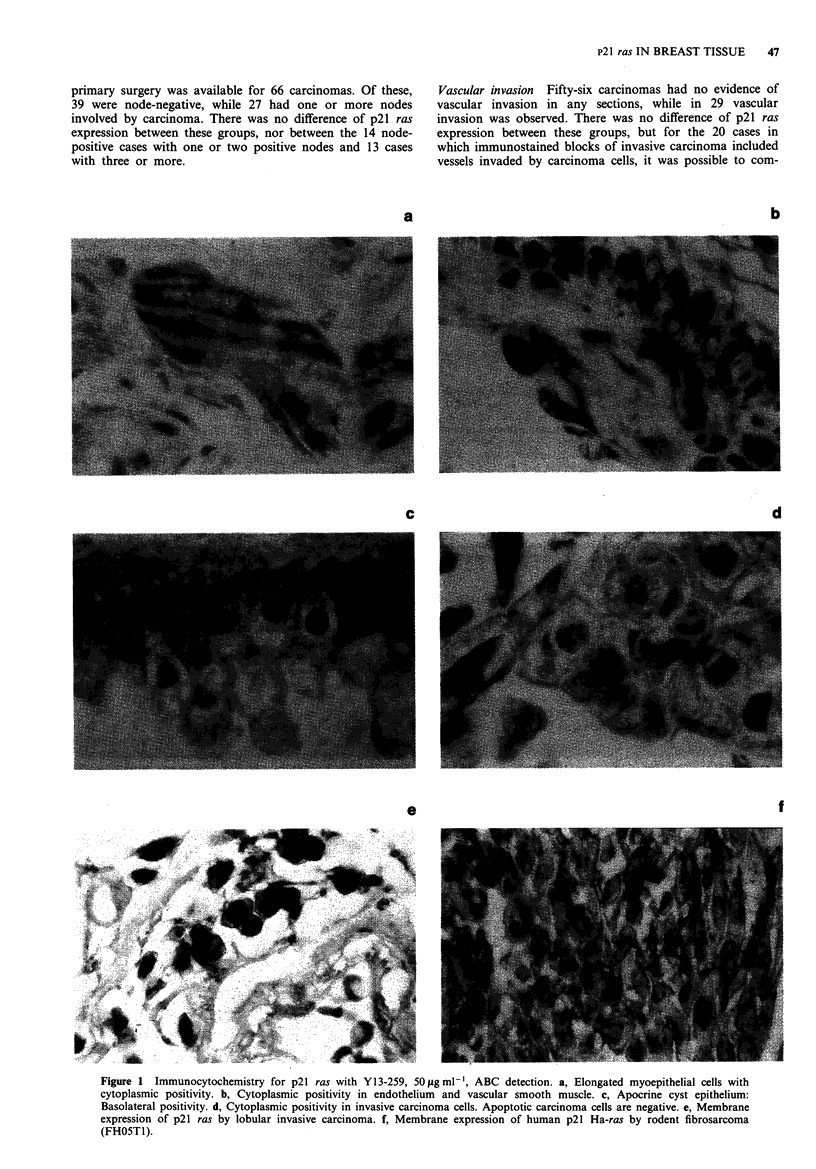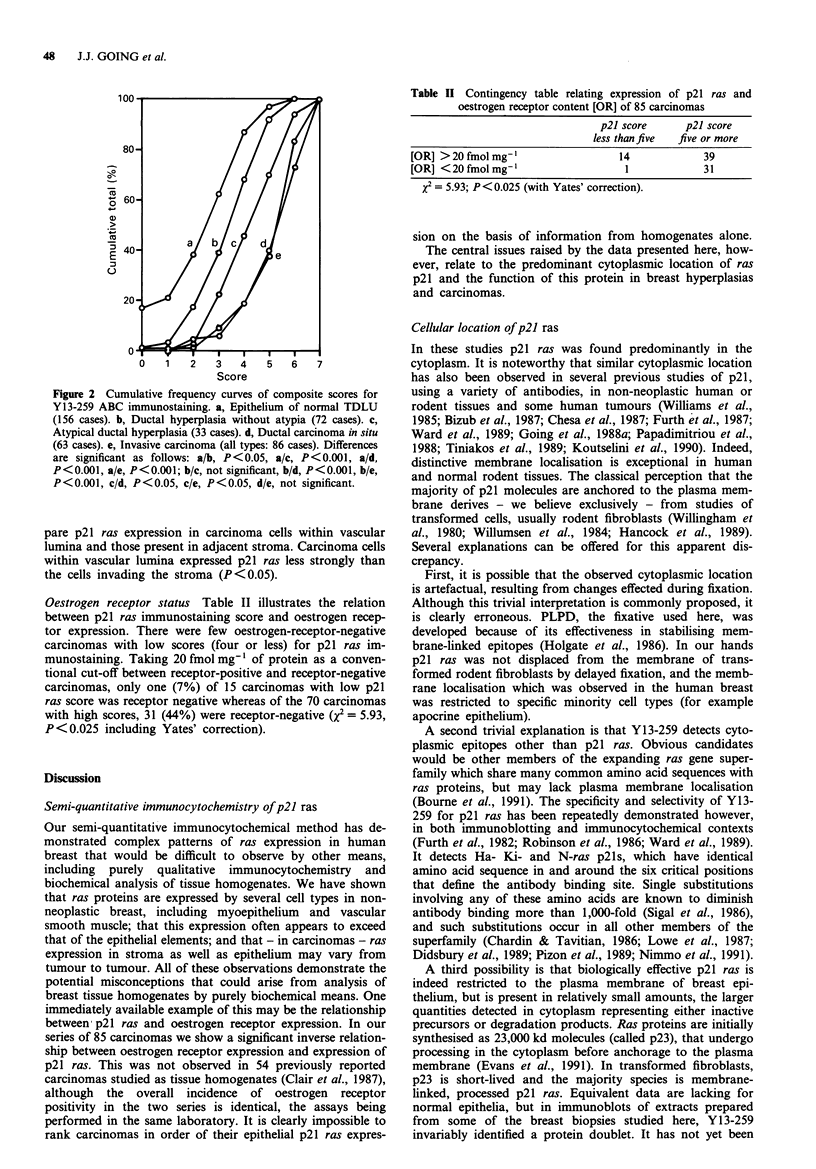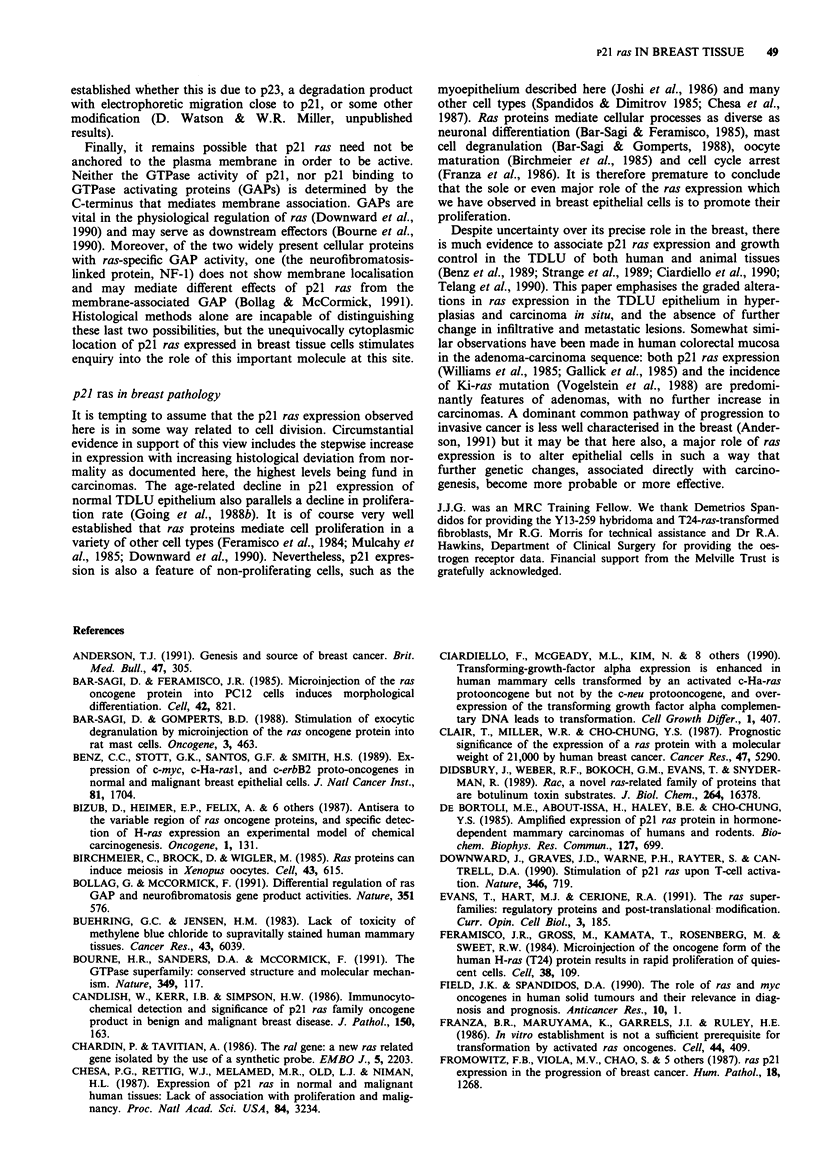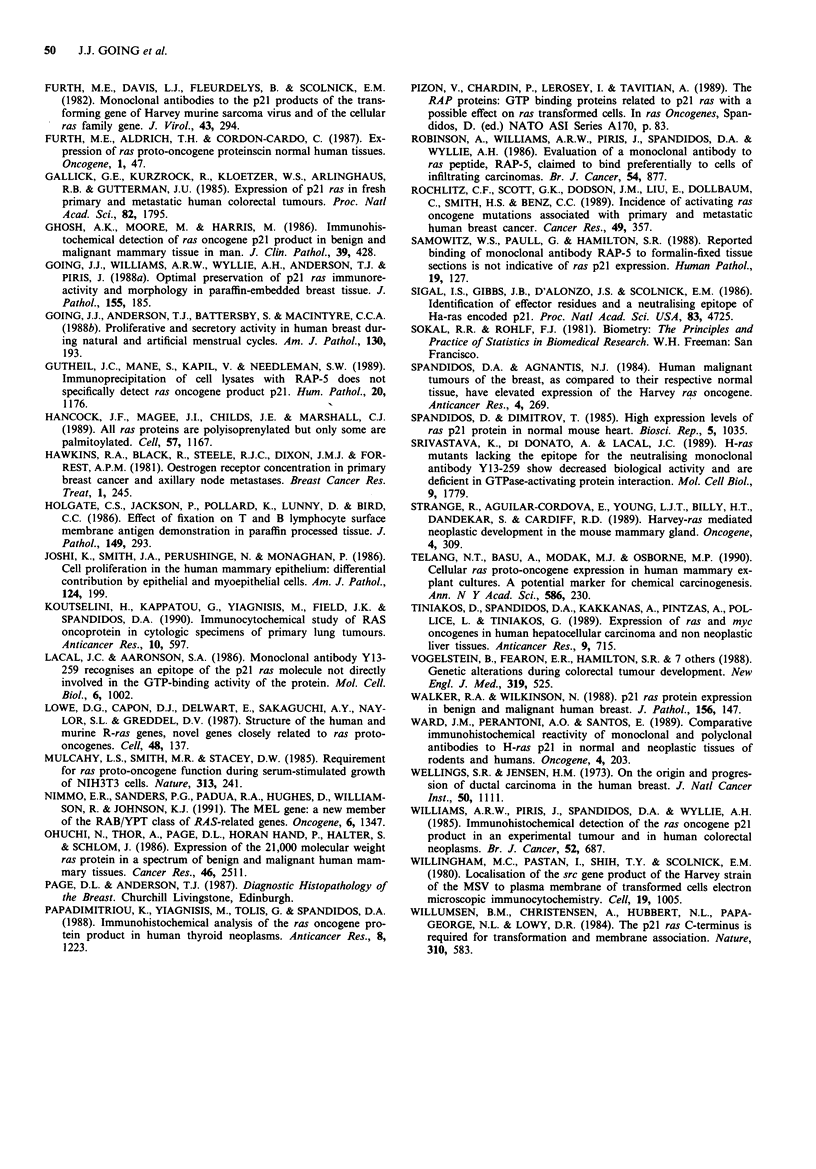# Ras p21 in breast tissue: associations with pathology and cellular localisation.

**DOI:** 10.1038/bjc.1992.9

**Published:** 1992-01

**Authors:** J. J. Going, T. J. Anderson, A. H. Wyllie

**Affiliations:** Department of Pathology, University Medical School, Edinburgh, UK.

## Abstract

**Images:**


 © Macmillan Press Ltd., 1992

### *Ras* p21 in breast tissue: associations with pathology and cellular localisation

J.J. Going, T.J. Anderson & A.H. Wyllie

*Cancer Research Campaign Laboratories, Department of Pathology, University Medical School, Teviot Place, Edinburgh EH8 9AG, UK.*

**Summary** Immunocytochemistry with monoclonal antibody Y13-259 demonstrated p21 *ras* in paraffin sections of breast tissue from 171 women: 85 with invasive breast carcinoma, 14 with non-invasive carcinoma and 72 with benign changes only. Many different tissue elements contributed to *ras* expression. Semiquantitative assessment showed that intensity of immunostaining in the normal epithelium of large ducts, small extralobular ducts and terminal duct lobular units (TDLU) was usually exceeded by that of myoepithelial cells. Vascular smooth muscle and apocrine epithelium also stained strongly, but the flat epithelial cells lining cysts did not express detectable p21 *ras*. There was a progressive increase from normal epithelium through epithelial hyperplasia of usual type and atypical hyperplasia to carcinoma *in situ*, without further increase in invasive carcinoma. Expression in carcinomas was inversely related to oestrogen receptor content but independent of the prognosis-associated variables of size, histological type, vascular invasion or lymph node metastasis.

The role of *ras* genes in human mammary carcinogenesis remains undefined. *Ras* mutations are rare in breast carcinomas (Rochlitz *et al*., 1989) and gene amplification has not been recorded, but there are conflicting observations (reviewed by Field & Spandidos, 1990) of *ras* protein expression. Hyperexpression has often been recorded (Spandidos & Agnantis, 1984; Ohuchi *et al*., 1986; De Bortoli *et al*., 1985; Clair *et al*., 1987; Fromowitz *et al*., 1987), but some immunocytochemical studies with the antibodies RAP-5 (Ghosh *et al*., 1986) and Y13-259 (Candlish *et al*., 1986; Walker & Wilkinson, 1988) have found no increased reactivity in carcinomas compared with benign parenchyma. Walker and Wilkinson (1988) suggested that expression in carcinomas was actually less than in normal parenchyma. Some of the discrepancies may reflect methodological differences. Conclusions from immunocytochemical data, for example, have been clouded by the now proven nonspecificity of RAP-5 (Robinson *et al*., 1986; Rochlitz *et al*., 1988; Samowitz *et al*., 1988; Gutheil *et al*., 1989). Interpretation of biochemical measurements of *ras* mRNA or protein is confounded by inclusion of many different cell types in the tissue homogenates analysed.

In this study we have used the pan-*ras* antibody Y13-259 (Furth *et al*., 1982). It is a well characterised rat monoclonal $IgG_1$, (Lacal *et al*., 1986), and when bound to its epitope, blocks the interaction of p21 *ras* with its potential downstream effector molecule, GTPase activating protein (GAP) (Srivastava *et al*., 1989). This epitope does not survive conventional aldehyde fixation well, but cryostat sections fixed in acetone (which conserves the epitope) are unsuitable for critical assessment of breast histology. Excellent preservation of morphology and Y13-259 immunoreactivity are obtained, however, in paraffin-embedded tissue fixed in periodate-lysine-paraformaldehyde (PLP) and its dichromate derivative (PLPD) (Going *et al*., 1988*a*). These fixatives preserve membrane localisation of p21 *ras* in transformed rodent fibroblasts expressing mutant p21 *ras*. No studies of human carcinoma have so far demonstrated the expected localisation of p21 *ras* to the plasma membrane (Going *et al*., 1988*a*; Williams *et al*., 1985; Chesa *et al*., 1987; Walker & Wilkinson, 1988). We therefore examined the cellular location of p21 *ras* in normal, hyperplastic and neoplastic breast tissue from 171 women, and used a semiquantitative scoring system to compare its staining intensity in immunocytochemical preparations that clearly display the different cell types and pathological states.

### Materials and methods

### *Patients and tissue selection*

One hundred and seventy-one women treated at the Breast Unit, Longmore Hospital, Edinburgh, formed the study population. Those who had received prior chemotherapy, radiotherapy, or endocrine manipulation by surgery or drugs were excluded. Three-mm slices were prepared from biopsy and mastectomy specimens collected immediately onto ice. Blocks were selected by naked-eye or dissecting-microscope inspection before or after supravital staining with methylene blue in ice-cold nutrient medium (Buehring & Jensen, 1983) to identify areas of abnormal parenchyma.

### *Fixation and processing*

Tissues were fixed in periodate-lysine-paraformaldehyde-dichromate (PLPD), processed to paraffin, and 4 μm sections immunostained with monoclonal antibody Y13-259 (obtainable from Oncogene Science) and an avidin-biotin detection system (Dako), together with appropriate controls (Going *et al*., 1988*a*). Morphological assessment of haematoxylin and eosin sections, including the diagnosis of special types of carcinoma, was by published criteria (Page & Anderson, 1987) and included comparison with diagnostic material fixed in buffered formaldehyde.

### *Scoring*

*Intensity* of immunostaining was scored 0-3 (negative-strong positive). *Extent* of immunostaining within a cellular population was scored 1-4 (0-25% positive = 1; 25-50% = 2; 50-75% = 3; 75-100% = 4). Intensity scores of 2 and 3 were added to the extent score to give a single *composite score*. In forming this composite score equivocally positive staining (intensity scoring 1) was ignored (Table I) to avoid giving undue weight to populations in which positivity was doubtful. For each section, composite scores were obtained separately for individual cell types (e.g. epithelium, myoepithelium, stromal fibroblasts) and, as appropriate, for each diagnostic category (e.g. normal, typical hyperplasia, atypical hyperplasia, carcinoma *in situ* etc).

Correspondence: J.J. Going, Department of Pathology, Glasgow Royal Infirmary, Castle Street, Glasgow G4 0SF, UK.
Received 1 February 1991; and in revised form 23 September 1991.

**Table I** Combination of extent and intensity scores for p21 *ras* immunocytochemistry to give an overall score (0–7)

| | | *Extent score* | | | |
|---|---|---|---|---|---|
| | | 1 | 2 | 3 | 4 |
| *Intensity* | 0 | – | – | – | 0 |
| *Score* | 1 | 1 | 1 | 2 | 2 |
| | 2 | 3 | 4 | 5 | 6 |
| | 3 | 4 | 5 | 6 | 7 |

0 implies no staining at all, seven implies strong immunostaining of 75–100% of the cells in a defined population.

*Statistical analysis*

Scoring was validated in 42 randomly chosen cases by repetition without knowledge of previously assigned scores, yielding 311 pairs of scores for separate populations of parenchymal and stromal cells. Acceptable repeatability of scoring was obtained (Spearman's rank correlation coefficient = 0.75, $P<0.001$). There was no systematic bias between scoring runs, assessed by Wilcoxon's matched-pairs signed ranks test ($P>0.05$). Comparison of composite immunostaining scores was performed by the two-tailed two-sample Kolmogorov-Smirnov test. This non-parametric test uses the greatest difference between cumulative frequency distributions of the samples under comparison (Sokal & Rohlf, 1981). Accordingly, the semiquantitative analyses in this paper are displayed as cumulative frequency curves rather than the more familiar frequency distribution histograms. Such plots show, in the form of a continuous curve, the percentage of cases in the studied population with scores that fall on or below the values displayed on the horizontal axis. The highest score recorded in a population is indicated by the value at which the curve reaches 100%, and the median score by the 50% point. Thus, generally low-scoring populations are represented by curves situated to the left of the plot, reaching 50% and 100% at relatively low score values. Populations with high scores appear as curves shifted to the right. The Kolmogorov-Smirnov test measures the significance of this shift.

*Oestrogen receptor status*

Oestrogen receptors were measured as previously described (Hawkins *et al.*, 1981) in homogenates made from tissue taken adjacent to the site selected for histology.

### Results

*Normal breast*

p21 *ras* expression in normal epithelium was weak and heterogenous. On semiquantitative assessment there was no difference in expression between ductules of terminal duct lobular units (TDLU), small extra-lobular ducts and larger ducts, and as both ductal and lobular carcinomas are thought to arise from TDLU (Wellings & Jensen, 1973), the epithelium of TDLU was selected to represent normal parenchyma in subsequent comparisons. Expression was the same in morphologically normal TDLU of women with cancer and women with benign changes only, but TDLU of women younger than 45 (the median age in our study population) expressed p21 *ras* more strongly than those of women aged 45 or over ($P<0.01$).

Myoepithelial cells in common with epithelial cells showed no difference in expression between large ducts, small ducts and TDLU, but overall expression in myoepithelial cells was consistently stronger than in epithelial cells ($P<0.001$; Figure 1a). Immunoreactivity with Y13-259 was also strong in vascular smooth muscle, and occasionally in normal endothelial cells (Figure 1b).

*Epithelium lining cysts*

Apocrine cyst epithelium was usually positive for p21 *ras* immunostaining, but some different patterns of *ras* expression were seen. In most cases there was distinct membrane localisation, usually apical, but in some cases baso-lateral (Figure 1c). In contrast the flattened simple epithelium of many cysts was consistently negative for p21 *ras* ($P<0.0001$).

*Hyperplastic and atypical epithelium*

There was progressively stronger and more extensive p21 *ras* expression from normal through hyperplastic and atypical epithelium to non-invasive carcinoma. Figure 2 presents p21 *ras* score data for usual and atypical ductal hyperplasias as well as normal TDLU, ductal carcinoma *in situ* (DCIS) and invasive carcinomas. The *P* values of differences between populations are listed in the caption. A similar progressive increase was seen from normal lobules through atypical lobular hyperplasia to lobular carcinoma *in situ*, but the numbers were too small for useful separate statistical analysis.

*Carcinomas*

Immunostaining for p21 *ras* was consistently strong in carcinomas. There was no difference in extent and intensity of immunostaining between non-invasive and invasive carcinoma, whether ductal or lobular. Expression of p21 *ras* was almost invariably stronger in carcinomas than in benign epithelium from the same patient. Some heterogeneity of expression was seen, with weakly staining or negative carcinoma cells adjacent to strongly expressing cells, but in many cases, uniform strong expression was observed in almost all malignant cells. One carcinoma only was devoid of detectable p21 *ras* while adjacent benign parenchyma was positive. Stromal cells of carcinomas were also consistently positive for p21 *ras*. Although not as strongly positive as carcinoma cells ($P<0.001$), they stained much more strongly than stromal cells of normal TDLU ($P<0.0001$).

In many carcinomas in which p21 *ras* was strongly expressed, cells undergoing apoptosis and cells in areas of confluent necrosis lost Y13-259 immunoreactivity (Figure 1d). This was also true for cells retaining some nuclear chromatin at the edge of areas of confluent necrosis, and there was usually a sharp transition from expression to non-expression.

*Cellular location of p21* ras

In almost all carcinomas, invasive or non-invasive, p21 *ras* expression was intracytoplasmic (Figure 1d). A few cases showed membrane as well as cytoplasmic positivity, and one lobular invasive carcinoma was unique in this human material in showing dominant membrane localisation (Figure 1e). By contrast, there was almost exclusive membrane expression in cells of a rodent fibroblast tumour expressing human Ha-*ras* (Going *et al.*, 1988*a*; Figure 1 f). In this tumour membrane localisation persisted even when fixation was deliberately delayed by up to 30 min (data not shown).

*Factors in clinicopathological correlation*

*Carcinoma size and histology* There were 18 invasive carcinomas of special histological type, including ten lobular, two medullary, two mucoid, three cribriform invasive and one tubular carcinoma. Sixty-seven were of no special type. No association was observed between histology and p21 *ras* expression. In particular, there was no correlation of p21 expression with types known to be associated with either poor or good prognosis. Similarly there was no correlation of p21 *ras* immunostaining with carcinoma diameter.

*Lymph node status* Accurate information from ipsilateral axillary clearance or four-node sampling at the time of primary surgery was available for 66 carcinomas. Of these, 39 were node-negative, while 27 had one or more nodes involved by carcinoma. There was no difference of p21 *ras* expression between these groups, nor between the 14 node-positive cases with one or two positive nodes and 13 cases with three or more.

*Vascular invasion* Fifty-six carcinomas had no evidence of vascular invasion in any sections, while in 29 vascular invasion was observed. There was no difference of p21 *ras* expression between these groups, but for the 20 cases in which immunostained blocks of invasive carcinoma included vessels invaded by carcinoma cells, it was possible to com-

**Figure 1** Immunocytochemistry for p21 *ras* with Y13-259, 50 $\mu g\,ml^{-1}$, ABC detection. **a**, Elongated myoepithelial cells with cytoplasmic positivity. **b**, Cytoplasmic positivity in endothelium and vascular smooth muscle. **c**, Apocrine cyst epithelium: Basolateral positivity. **d**, Cytoplasmic positivity in invasive carcinoma cells. Apoptotic carcinoma cells are negative. **e**, Membrane expression of p21 *ras* by lobular invasive carcinoma. **f**, Membrane expression of human p21 Ha-*ras* by rodent fibrosarcoma (FH05T1).

**Figure 2** Cumulative frequency curves of composite scores for Y13-259 ABC immunostaining. **a**, Epithelium of normal TDLU (156 cases). **b**, Ductal hyperplasia without atypia (72 cases). **c**, Atypical ductal hyperplasia (33 cases). **d**, Ductal carcinoma *in situ* (63 cases). **e**, Invasive carcinoma (all types: 86 cases). Differences are significant as follows: **a/b**, $P<0.05$, **a/c**, $P<0.001$, **a/d**, $P<0.001$, **a/e**, $P<0.001$; **b/c**, not significant, **b/d**, $P<0.001$, **b/e**, $P<0.001$, **c/d**, $P<0.05$, **c/e**, $P<0.05$, **d/e**, not significant.

pare p21 *ras* expression in carcinoma cells within vascular lumina and those present in adjacent stroma. Carcinoma cells within vascular lumina expressed p21 *ras* less strongly than the cells invading the stroma ($P<0.05$).

*Oestrogen receptor status* Table II illustrates the relation between p21 *ras* immunostaining score and oestrogen receptor expression. There were few oestrogen-receptor-negative carcinomas with low scores (four or less) for p21 *ras* immunostaining. Taking 20 fmol mg$^{-1}$ of protein as a conventional cut-off between receptor-positive and receptor-negative carcinomas, only one (7%) of 15 carcinomas with low p21 *ras* score was receptor negative whereas of the 70 carcinomas with high scores, 31 (44%) were receptor-negative ($\chi^2 = 5.93$, $P<0.025$ including Yates' correction).

### Discussion

### *Semi-quantitative immunocytochemistry of p21* ras

Our semi-quantitative immunocytochemical method has demonstrated complex patterns of *ras* expression in human breast that would be difficult to observe by other means, including purely qualitative immunocytochemistry and biochemical analysis of tissue homogenates. We have shown that *ras* proteins are expressed by several cell types in non-neoplastic breast, including myoepithelium and vascular smooth muscle; that this expression often appears to exceed that of the epithelial elements; and that – in carcinomas – *ras* expression in stroma as well as epithelium may vary from tumour to tumour. All of these observations demonstrate the potential misconceptions that could arise from analysis of breast tissue homogenates by purely biochemical means. One immediately available example of this may be the relationship between p21 *ras* and oestrogen receptor expression. In our series of 85 carcinomas we show a significant inverse relationship between oestrogen receptor expression and expression of p21 *ras*. This was not observed in 54 previously reported carcinomas studied as tissue homogenates (Clair *et al.*, 1987), although the overall incidence of oestrogen receptor positivity in the two series is identical, the assays being performed in the same laboratory. It is clearly impossible to rank carcinomas in order of their epithelial p21 *ras* expression on the basis of information from homogenates alone.

**Table II** Contingency table relating expression of p21 *ras* and oestrogen receptor content [OR] of 85 carcinomas

| | *p21 score less than five* | *p21 score five or more* |
|---|---|---|
| [OR] >20 fmol mg$^{-1}$ | 14 | 39 |
| [OR] <20 fmol mg$^{-1}$ | 1 | 31 |

$\chi^2 = 5.93$; $P<0.025$ (with Yates' correction).

The central issues raised by the data presented here, however, relate to the predominant cytoplasmic location of *ras* p21 and the function of this protein in breast hyperplasias and carcinomas.

### *Cellular location of p21* ras

In these studies p21 *ras* was found predominantly in the cytoplasm. It is noteworthy that similar cytoplasmic location has also been observed in several previous studies of p21, using a variety of antibodies, in non-neoplastic human or rodent tissues and some human tumours (Williams *et al.*, 1985; Bizub *et al.*, 1987; Chesa *et al.*, 1987; Furth *et al.*, 1987; Ward *et al.*, 1989; Going *et al.*, 1988*a*; Papadimitriou *et al.*, 1988; Tiniakos *et al.*, 1989; Koutselini *et al.*, 1990). Indeed, distinctive membrane localisation is exceptional in human and normal rodent tissues. The classical perception that the majority of p21 molecules are anchored to the plasma membrane derives – we believe exclusively – from studies of transformed cells, usually rodent fibroblasts (Willingham *et al.*, 1980; Willumsen *et al.*, 1984; Hancock *et al.*, 1989). Several explanations can be offered for this apparent discrepancy.

First, it is possible that the observed cytoplasmic location is artefactual, resulting from changes effected during fixation. Although this trivial interpretation is commonly proposed, it is clearly erroneous. PLPD, the fixative used here, was developed because of its effectiveness in stabilising membrane-linked epitopes (Holgate *et al.*, 1986). In our hands p21 *ras* was not displaced from the membrane of transformed rodent fibroblasts by delayed fixation, and the membrane localisation which was observed in the human breast was restricted to specific minority cell types (for example apocrine epithelium).

A second trivial explanation is that Y13-259 detects cytoplasmic epitopes other than p21 *ras*. Obvious candidates would be other members of the expanding *ras* gene superfamily which share many common amino acid sequences with *ras* proteins, but may lack plasma membrane localisation (Bourne *et al.*, 1991). The specificity and selectivity of Y13-259 for p21 *ras* has been repeatedly demonstrated however, in both immunoblotting and immunocytochemical contexts (Furth *et al.*, 1982; Robinson *et al.*, 1986; Ward *et al.*, 1989). It detects Ha- Ki- and N-*ras* p21s, which have identical amino acid sequence in and around the six critical positions that define the antibody binding site. Single substitutions involving any of these amino acids are known to diminish antibody binding more than 1,000-fold (Sigal *et al.*, 1986), and such substitutions occur in all other members of the superfamily (Chardin & Tavitian, 1986; Lowe *et al.*, 1987; Didsbury *et al.*, 1989; Pizon *et al.*, 1989; Nimmo *et al.*, 1991).

A third possibility is that biologically effective p21 *ras* is indeed restricted to the plasma membrane of breast epithelium, but is present in relatively small amounts, the larger quantities detected in cytoplasm representing either inactive precursors or degradation products. *Ras* proteins are initially synthesised as 23,000 kd molecules (called p23), that undergo processing in the cytoplasm before anchorage to the plasma membrane (Evans *et al.*, 1991). In transformed fibroblasts, p23 is short-lived and the majority species is membrane-linked, processed p21 *ras*. Equivalent data are lacking for normal epithelia, but in immunoblots of extracts prepared from some of the breast biopsies studied here, Y13-259 invariably identified a protein doublet. It has not yet been established whether this is due to p23, a degradation product with electrophoretic migration close to p21, or some other modification (D. Watson & W.R. Miller, unpublished results).

Finally, it remains possible that p21 *ras* need not be anchored to the plasma membrane in order to be active. Neither the GTPase activity of p21, nor p21 binding to GTPase activating proteins (GAPs) is determined by the C-terminus that mediates membrane association. GAPs are vital in the physiological regulation of *ras* (Downward *et al.*, 1990) and may serve as downstream effectors (Bourne *et al.*, 1990). Moreover, of the two widely present cellular proteins with *ras*-specific GAP activity, one (the neurofibromatosis-linked protein, NF-1) does not show membrane localisation and may mediate different effects of p21 *ras* from the membrane-associated GAP (Bollag & McCormick, 1991). Histological methods alone are incapable of distinguishing these last two possibilities, but the unequivocally cytoplasmic location of p21 *ras* expressed in breast tissue cells stimulates enquiry into the role of this important molecule at this site.

### *p21* ras *in breast pathology*

It is tempting to assume that the p21 *ras* expression observed here is in some way related to cell division. Circumstantial evidence in support of this view includes the stepwise increase in expression with increasing histological deviation from normality as documented here, the highest levels being fund in carcinomas. The age-related decline in p21 expression of normal TDLU epithelium also parallels a decline in proliferation rate (Going *et al.*, 1988*b*). It is of course very well established that *ras* proteins mediate cell proliferation in a variety of other cell types (Feramisco *et al.*, 1984; Mulcahy *et al.*, 1985; Downward *et al.*, 1990). Nevertheless, p21 expression is also a feature of non-proliferating cells, such as the myoepithelium described here (Joshi *et al.*, 1986) and many other cell types (Spandidos & Dimitrov 1985; Chesa *et al.*, 1987). *Ras* proteins mediate cellular processes as diverse as neuronal differentiation (Bar-Sagi & Feramisco, 1985), mast cell degranulation (Bar-Sagi & Gomperts, 1988), oocyte maturation (Birchmeier *et al.*, 1985) and cell cycle arrest (Franza *et al.*, 1986). It is therefore premature to conclude that the sole or even major role of the *ras* expression which we have observed in breast epithelial cells is to promote their proliferation.

Despite uncertainty over its precise role in the breast, there is much evidence to associate p21 *ras* expression and growth control in the TDLU of both human and animal tissues (Benz *et al.*, 1989; Strange *et al.*, 1989; Ciardiello *et al.*, 1990; Telang *et al.*, 1990). This paper emphasises the graded alterations in *ras* expression in the TDLU epithelium in hyperplasias and carcinoma *in situ*, and the absence of further change in infiltrative and metastatic lesions. Somewhat similar observations have been made in human colorectal mucosa in the adenoma-carcinoma sequence: both p21 *ras* expression (Williams *et al.*, 1985; Gallick *et al.*, 1985) and the incidence of Ki-*ras* mutation (Vogelstein *et al.*, 1988) are predominantly features of adenomas, with no further increase in carcinomas. A dominant common pathway of progression to invasive cancer is less well characterised in the breast (Anderson, 1991) but it may be that here also, a major role of *ras* expression is to alter epithelial cells in such a way that further genetic changes, associated directly with carcinogenesis, become more probable or more effective.

J.J.G. was an MRC Training Fellow. We thank Demetrios Spandidos for providing the Y13-259 hybridoma and T24-*ras*-transformed fibroblasts, Mr R.G. Morris for technical assistance and Dr R.A. Hawkins, Department of Clinical Surgery for providing the oestrogen receptor data. Financial support from the Melville Trust is gratefully acknowledged.

### References

ANDERSON, T.J. (1991). Genesis and source of breast cancer. *Brit. Med. Bull.*, **47**, 305.

BAR-SAGI, D. & FERAMISCO, J.R. (1985). Microinjection of the *ras* oncogene protein into PC12 cells induces morphological differentiation. *Cell*, **42**, 821.

BAR-SAGI, D. & GOMPERTS, B.D. (1988). Stimulation of exocytic degranulation by microinjection of the *ras* oncogene protein into rat mast cells. *Oncogene*, **3**, 463.

BENZ, C.C., STOTT, G.K., SANTOS, G.F. & SMITH, H.S. (1989). Expression of c-*myc*, c-Ha-*ras*1, and c-*erb*B2 proto-oncogenes in normal and malignant breast epithelial cells. *J. Natl Cancer Inst.*, **81**, 1704.

BIZUB, D., HEIMER, E.P., FELIX, A. & 6 others (1987). Antisera to the variable region of *ras* oncogene proteins, and specific detection of H-*ras* expression an experimental model of chemical carcinogenesis. *Oncogene*, **1**, 131.

BIRCHMEIER, C., BROCK, D. & WIGLER, M. (1985). *Ras* proteins can induce meiosis in *Xenopus* oocytes. *Cell*, **43**, 615.

BOLLAG, G. & MCCORMICK, F. (1991). Differential regulation of ras GAP and neurofibromatosis gene product activities. *Nature*, **351** 576.

BUEHRING, G.C. & JENSEN, H.M. (1983). Lack of toxicity of methylene blue chloride to supravitally stained human mammary tissues. *Cancer Res.*, **43**, 6039.

BOURNE, H.R., SANDERS, D.A. & MCCORMICK, F. (1991). The GTPase superfamily: conserved structure and molecular mechanism. *Nature*, **349**, 117.

CANDLISH, W., KERR, I.B. & SIMPSON, H.W. (1986). Immunocytochemical detection and significance of p21 *ras* family oncogene product in benign and malignant breast disease. *J. Pathol.*, **150**, 163.

CHARDIN, P. & TAVITIAN, A. (1986). The *ral* gene: a new *ras* related gene isolated by the use of a synthetic probe. *EMBO J.*, **5**, 2203.

CHESA, P.G., RETTIG, W.J., MELAMED, M.R., OLD, L.J. & NIMAN, H.L. (1987). Expression of p21 *ras* in normal and malignant human tissues: Lack of association with proliferation and malignancy. *Proc. Natl Acad. Sci. USA*, **84**, 3234.

CIARDIELLO, F., MCGEADY, M.L., KIM, N. & 8 others (1990). Transforming-growth-factor alpha expression is enhanced in human mammary cells transformed by an activated c-Ha-*ras* protooncogene but not by the c-*neu* protooncogene, and overexpression of the transforming growth factor alpha complementary DNA leads to transformation. *Cell Growth Differ.*, **1**, 407.

CLAIR, T., MILLER, W.R. & CHO-CHUNG, Y.S. (1987). Prognostic significance of the expression of a *ras* protein with a molecular weight of 21,000 by human breast cancer. *Cancer Res.*, **47**, 5290.

DIDSBURY, J., WEBER, R.F., BOKOCH, G.M., EVANS, T. & SNYDERMAN, R. (1989). *Rac*, a novel *ras*-related family of proteins that are botulinum toxin substrates. *J. Biol. Chem.*, **264**, 16378.

DE BORTOLI, M.E., ABOUT-ISSA, H., HALEY, B.E. & CHO-CHUNG, Y.S. (1985). Amplified expression of p21 *ras* protein in hormone-dependent mammary carcinomas of humans and rodents. *Biochem. Biophys. Res. Commun.*, **127**, 699.

DOWNWARD, J., GRAVES, J.D., WARNE, P.H., RAYTER, S. & CANTRELL, D.A. (1990). Stimulation of p21 *ras* upon T-cell activation. *Nature*, **346**, 719.

EVANS, T., HART, M.J. & CERIONE, R.A. (1991). The *ras* superfamilies: regulatory proteins and post-translational modification. *Curr. Opin. Cell Biol.*, **3**, 185.

FERAMISCO, J.R., GROSS, M., KAMATA, T., ROSENBERG, M. & SWEET, R.W. (1984). Microinjection of the oncogene form of the human H-*ras* (T24) protein results in rapid proliferation of quiescent cells. *Cell*, **38**, 109.

FIELD, J.K. & SPANDIDOS, D.A. (1990). The role of *ras* and *myc* oncogenes in human solid tumours and their relevance in diagnosis and prognosis. *Anticancer Res.*, **10**, 1.

FRANZA, B.R., MARUYAMA, K., GARRELS, J.I. & RULEY, H.E. (1986). *In vitro* establishment is not a sufficient prerequisite for transformation by activated *ras* oncogenes. *Cell*, **44**, 409.

FROMOWITZ, F.B., VIOLA, M.V., CHAO, S. & 5 others (1987). *ras* p21 expression in the progression of breast cancer. *Hum. Pathol.*, **18**, 1268.

FURTH, M.E., DAVIS, L.J., FLEURDELYS, B. & SCOLNICK, E.M. (1982). Monoclonal antibodies to the p21 products of the transforming gene of Harvey murine sarcoma virus and of the cellular *ras* family gene. *J. Virol.*, **43**, 294.

FURTH, M.E., ALDRICH, T.H. & CORDON-CARDO, C. (1987). Expression of *ras* proto-oncogene proteinscin normal human tissues. *Oncogene*, **1**, 47.

GALLICK, G.E., KURZROCK, R., KLOETZER, W.S., ARLINGHAUS, R.B. & GUTTERMAN, J.U. (1985). Expression of p21 *ras* in fresh primary and metastatic human colorectal tumours. *Proc. Natl Acad. Sci.*, **82**, 1795.

GHOSH, A.K., MOORE, M. & HARRIS, M. (1986). Immunohistochemical detection of *ras* oncogene p21 product in benign and malignant mammary tissue in man. *J. Clin. Pathol.*, **39**, 428.

GOING, J.J., WILLIAMS, A.R.W., WYLLIE, A.H., ANDERSON, T.J. & PIRIS, J. (1988*a*). Optimal preservation of p21 *ras* immunoreactivity and morphology in paraffin-embedded breast tissue. *J. Pathol.*, **155**, 185.

GOING, J.J., ANDERSON, T.J., BATTERSBY, S. & MACINTYRE, C.C.A. (1988*b*). Proliferative and secretory activity in human breast during natural and artificial menstrual cycles. *Am. J. Pathol.*, **130**, 193.

GUTHEIL, J.C., MANE, S., KAPIL, V. & NEEDLEMAN, S.W. (1989). Immunoprecipitation of cell lysates with RAP-5 does not specifically detect *ras* oncogene product p21. *Hum. Pathol.*, **20**, 1176.

HANCOCK, J.F., MAGEE, J.I., CHILDS, J.E. & MARSHALL, C.J. (1989). All *ras* proteins are polyisoprenylated but only some are palmitoylated. *Cell*, **57**, 1167.

HAWKINS, R.A., BLACK, R., STEELE, R.J.C., DIXON, J.M.J. & FORREST, A.P.M. (1981). Oestrogen receptor concentration in primary breast cancer and axillary node metastases. *Breast Cancer Res. Treat*, **1**, 245.

HOLGATE, C.S., JACKSON, P., POLLARD, K., LUNNY, D. & BIRD, C.C. (1986). Effect of fixation on T and B lymphocyte surface membrane antigen demonstration in paraffin processed tissue. *J. Pathol.*, **149**, 293.

JOSHI, K., SMITH, J.A., PERUSHINGE, N. & MONAGHAN, P. (1986). Cell proliferation in the human mammary epithelium: differential contribution by epithelial and myoepithelial cells. *Am. J. Pathol.*, **124**, 199.

KOUTSELINI, H., KAPPATOU, G., YIAGNISIS, M., FIELD, J.K. & SPANDIDOS, D.A. (1990). Immunocytochemical study of RAS oncoprotein in cytologic specimens of primary lung tumours. *Anticancer Res.*, **10**, 597.

LACAL, J.C. & AARONSON, S.A. (1986). Monoclonal antibody Y13-259 recognises an epitope of the p21 *ras* molecule not directly involved in the GTP-binding activity of the protein. *Mol. Cell. Biol.*, **6**, 1002.

LOWE, D.G., CAPON, D.J., DELWART, E., SAKAGUCHI, A.Y., NAYLOR, S.L. & GREDDEL, D.V. (1987). Structure of the human and murine R-*ras* genes, novel genes closely related to *ras* proto-oncogenes. *Cell*, **48**, 137.

MULCAHY, L.S., SMITH, M.R. & STACEY, D.W. (1985). Requirement for *ras* proto-oncogene function during serum-stimulated growth of NIH3T3 cells. *Nature*, **313**, 241.

NIMMO, E.R., SANDERS, P.G., PADUA, R.A., HUGHES, D., WILLIAMSON, R. & JOHNSON, K.J. (1991). The MEL gene: a new member of the RAB/YPT class of *RAS*-related genes. *Oncogene*, **6**, 1347.

OHUCHI, N., THOR, A., PAGE, D.L., HORAN HAND, P., HALTER, S. & SCHLOM, J. (1986). Expression of the 21,000 molecular weight *ras* protein in a spectrum of benign and malignant human mammary tissues. *Cancer Res.*, **46**, 2511.

PAGE, D.L. & ANDERSON, T.J. (1987). *Diagnostic Histopathology of the Breast*. Churchill Livingstone, Edinburgh.

PAPADIMITRIOU, K., YIAGNISIS, M., TOLIS, G. & SPANDIDOS, D.A. (1988). Immunohistochemical analysis of the *ras* oncogene protein product in human thyroid neoplasms. *Anticancer Res.*, **8**, 1223.

PIZON, V., CHARDIN, P., LEROSEY, I. & TAVITIAN, A. (1989). The *RAP* proteins: GTP binding proteins related to p21 *ras* with a possible effect on *ras* transformed cells. In *ras Oncogenes*, Spandidos, D. (ed.) NATO ASI Series A170, p. 83.

ROBINSON, A., WILLIAMS, A.R.W., PIRIS, J., SPANDIDOS, D.A. & WYLLIE, A.H. (1986). Evaluation of a monoclonal antibody to *ras* peptide, RAP-5, claimed to bind preferentially to cells of infiltrating carcinomas. *Br. J. Cancer*, **54**, 877.

ROCHLITZ, C.F., SCOTT, G.K., DODSON, J.M., LIU, E., DOLLBAUM, C., SMITH, H.S. & BENZ, C.C. (1989). Incidence of activating *ras* oncogene mutations associated with primary and metastatic human breast cancer. *Cancer Res.*, **49**, 357.

SAMOWITZ, W.S., PAULL, G. & HAMILTON, S.R. (1988). Reported binding of monoclonal antibody RAP-5 to formalin-fixed tissue sections is not indicative of *ras* p21 expression. *Human Pathol.*, **19**, 127.

SIGAL, I.S., GIBBS, J.B., D'ALONZO, J.S. & SCOLNICK, E.M. (1986). Identification of effector residues and a neutralising epitope of Ha-ras encoded p21. *Proc. Natl Acad. Sci. USA*, **83**, 4725.

SOKAL, R.R. & ROHLF, F.J. (1981). Biometry: *The Principles and Practice of Statistics in Biomedical Research*. W.H. Freeman: San Francisco.

SPANDIDOS, D.A. & AGNANTIS, N.J. (1984). Human malignant tumours of the breast, as compared to their respective normal tissue, have elevated expression of the Harvey *ras* oncogene. *Anticancer Res.*, **4**, 269.

SPANDIDOS, D. & DIMITROV, T. (1985). High expression levels of *ras* p21 protein in normal mouse heart. *Biosci. Rep.*, **5**, 1035.

SRIVASTAVA, K., DI DONATO, A. & LACAL, J.C. (1989). H-*ras* mutants lacking the epitope for the neutralising monoclonal antibody Y13-259 show decreased biological activity and are deficient in GTPase-activating protein interaction. *Mol. Cell Biol.*, **9**, 1779.

STRANGE, R., AGUILAR-CORDOVA, E., YOUNG, L.J.T., BILLY, H.T., DANDEKAR, S. & CARDIFF, R.D. (1989). Harvey-*ras* mediated neoplastic development in the mouse mammary gland. *Oncogene*, **4**, 309.

TELANG, N.T., BASU, A., MODAK, M.J. & OSBORNE, M.P. (1990). Cellular *ras* proto-oncogene expression in human mammary explant cultures. A potential marker for chemical carcinogenesis. *Ann. N Y Acad. Sci.*, **586**, 230.

TINIAKOS, D., SPANDIDOS, D.A., KAKKANAS, A., PINTZAS, A., POLLICE, L. & TINIAKOS, G. (1989). Expression of *ras* and *myc* oncogenes in human hepatocellular carcinoma and non neoplastic liver tissues. *Anticancer Res.*, **9**, 715.

VOGELSTEIN, B., FEARON, E.R., HAMILTON, S.R. & 7 others (1988). Genetic alterations during colorectal tumour development. *New Engl. J. Med.*, **319**, 525.

WALKER, R.A. & WILKINSON, N. (1988). p21 *ras* protein expression in benign and malignant human breast. *J. Pathol.*, **156**, 147.

WARD, J.M., PERANTONI, A.O. & SANTOS, E. (1989). Comparative immunohistochemical reactivity of monoclonal and polyclonal antibodies to H-*ras* p21 in normal and neoplastic tissues of rodents and humans. *Oncogene*, **4**, 203.

WELLINGS, S.R. & JENSEN, H.M. (1973). On the origin and progression of ductal carcinoma in the human breast. *J. Natl Cancer Inst.*, **50**, 1111.

WILLIAMS, A.R.W., PIRIS, J., SPANDIDOS, D.A. & WYLLIE, A.H. (1985). Immunohistochemical detection of the *ras* oncogene p21 product in an experimental tumour and in human colorectal neoplasms. *Br. J. Cancer*, **52**, 687.

WILLINGHAM, M.C., PASTAN, I., SHIH, T.Y. & SCOLNICK, E.M. (1980). Localisation of the *src* gene product of the Harvey strain of the MSV to plasma membrane of transformed cells electron microscopic immunocytochemistry. *Cell*, **19**, 1005.

WILLUMSEN, B.M., CHRISTENSEN, A., HUBBERT, N.L., PAPAGEORGE, N.L. & LOWY, D.R. (1984). The p21 *ras* C-terminus is required for transformation and membrane association. *Nature*, **310**, 583.